# Male sterility and hybrid breeding in soybean

**DOI:** 10.1007/s11032-023-01390-4

**Published:** 2023-05-25

**Authors:** Xiaolong Fang, Yanyan Sun, Jinhong Li, Meina Li, Chunbao Zhang

**Affiliations:** 1 Guangdong Provincial Key Laboratory of Plant Adaptation and Molecular Design, Innovative Center of Molecular Genetics and Evolution, School of Life Sciences, Guangzhou University, Guangzhou, 510006 Guangdong China; 2 Key Laboratory of Hybrid Soybean Breeding of the Ministry of Agriculture and Rural Affairs, Soybean Research Institute, Jilin Academy of Agricultural Sciences, Changchun, 130033 Jilin China

**Keywords:** Soybean, Hybrid breeding, Male sterility, Cytoplasmic male sterility (CMS), Genic male sterility (GMS)

## Abstract

Hybrid breeding can help us to meet the challenge of feeding a growing world population with limited agricultural land. The demand for soybean is expected to grow; however, the hybrid soybean is still in the process of commercialization even though considerable progress has been made in soybean genome and genetic studies in recent years. Here, we summarize recent advances in male sterility-based breeding programs and the current status of hybrid soybean breeding. A number of male-sterile lines with cytoplasmic male sterility (CMS), genic-controlled photoperiod/thermo-sensitive male sterility, and stable nuclear male sterility (GMS) have been identified in soybean. More than 40 hybrid soybean varieties have been bred using the CMS three-line hybrid system and the cultivation of hybrid soybean is still under way. The key to accelerating hybrid soybean breeding is to increase the out-crossing rate in an economical way. This review outlines current problems with the hybrid soybean breeding systems and explores the current efforts to make the hybrid soybean a commercial success.

## Introduction

The area of soybean (*Glycine max*) cultivation in the world has expanded by more than 900% since the 1960s in North and South America, due to its significant roles in animal feeding and human nutrition (http://www.fao.org/faostat/en/#home). However, soybean yield per unit area has not changed significantly compared with rice, wheat, and maize, suggesting the lack of a true Green Revolution in soybean breeding (Liu, et al. 2020a). Soybean yield is determined by both the total number of nodes and the number of pods per node, therefore the yield increase cannot be achieved in soybean by simply adoption of the shorter varieties. There are several options to increase soybean yields, and hybrid breeding hold the greatest potential to boost yield.

Soybean is an autogamous legume species, and male sterility line is a prerequisites for commercially available hybrid breeding and large quantities of seed production. An earlier heterosis test demonstrated that significant yield increases could be achieved in soybean; the heterozygous F_1_ plants of 248 combinations yielded 20% more than their parental lines among the 1123 combinations that were tested (Sun, et al. 1999; Palmer, et al. 2001). However, hybrid breeding in soybean has received limited attention in contrast to maize and rice. Male-sterile female lines with cytoplasmic male sterility (CMS) or genic-controlled photoperiod/thermo-sensitive male sterility (P/TGMS) have been extensively used for many years in maize and rice (Chen and Liu, 2014; Wan, et al. 2019). Hybrid rice in China covers 50–60% of the total rice cultivation fields, which contributed greatly to rice yield and ensure food security (Kim and Zhang, 2018; Liao, et al. 2021).

Male sterility lines are available in soybean, and the first soybean CMS line was reported under the US patents No. 4545146 in Davis (1985). Since then, no further information on this CMS line has been reported. Considerable research on soybean CMS lines has been conducted in China since the early 80s of the last century (Sun, et al. 1994a; Palmer, et al. 2001). To date, more than 40 hybrid soybean varieties have been bred and approved in China after several generations of researchers with more than 40 years of efforts, and more than 30 invention patents and technical standards have been authorized for the use of new technologies and methods for soybean hybrid breeding (Sun, et al. 2021). However, the CMS genes and underlying molecular mechanisms are still unknown in soybean, which has restricted the development of commercial varieties.

With the explosion in genomic resources and the rapid development of molecular biology and technology, the biotechnology-based male-sterility (BMS) systems for hybrid breeding have been established in maize, rice and other crops and vegetables (Chang, et al. 2016; Wu, et al. 2016; Singh, et al. 2019). The BMS systems utilize nuclear male sterility to propagate the pure nuclear male sterile seeds on a large scale, which not only make the climate change not a threat to the pure hybrid seed production anymore, but also unlock the potential for breeding superior hybrids through expanding the parental germplasm pool. In soybean, the nuclear male sterile mutants *ms4*, *ms1*, *ms6*, and *ms3* have been cloned in recent years (Thu, et al. 2019; Fang, et al. 2021; Jiang, et al. 2021; Nadeem, et al. 2021; Yu, et al. 2021; Hou, et al. 2022). To speed up the large-scale commercial cultivation of hybrid soybean, it is time to consider where to put the investments, should we continue to count on the three-line hybrid system and looking for the ideal maintainer and restorer lines, or we can rely on the BMS systems to realize the commercialization of hybrid soybean. In this review, we try to cover recent advances in cytoplasmic-nuclear and nuclear male sterility systems in soybean to see if the technological breakthroughs will make us to succeed in hybrid soybean production.

## Male sterility in plant

Plant male sterility (MS) refers to the phenomenon that the stamen develops unnormal, losing the ability to produce the functionally active male gametes for fertilization. According to their phenotypic characteristics, Kaul (1988) divide MS into three categories including structural, sporogenous, and functional. Structural MS indicates that the stamen is either completely absent or abnormally formed, which results in the absence of pollen. Sporogenous MS indicates that the stamen is essentially morphological normal, but fail to produce functional microspores or pollen due to the failure of early microsporogenesis and late microgametogenesis. Functional MS indicates that the viable pollen is produced, but either cannot be released from the anther due to the absence of dehiscence or is unable to geminate on the stigma and to initiate fertilization.

On the other scheme, according to the origin of inheritance, two types of MS are distinguished: cytoplasmic male sterility (CMS) and nuclear or genic male sterility (GMS). CMS is co-controlled by the nuclear and cytoplasmic genes, while GMS is controlled by the nuclear genes alone. CMS is widely spread in the higher plants and more than 300 species possess CMS were reported up to now (Liu, et al. 2001). The CMS is the result of the incompatibility between nuclear and mitochondrial gene products and there are several ways to generate CMS, including wide/inter-specific hybridization, protoplasmic fusion, induced mutations and genetic engineering (Bohra, et al. 2016). GMS is derived from the changes in the structure and function of nuclear genes, most of which are caused by natural variation and can also be achieved by physical and chemical mutagenesis. Mostly, the fertility of a GMS line is controlled by a recessive gene, and rarely by a dominant gene.

## Cytoplasmic male sterility (CMS) system

*CMS/Rf* (restorer-of-fertility) system, also known as the three-line hybrid system, comprises a cytoplasmic male sterile line, a maintainer line, and a restorer line. The sterile line contains a cytoplasmic male sterile gene, while lacks a nuclear restorer gene (Schnable and Wise, 1998), which is characterized by sterile pollen and unable to produce progeny by self-inbreeding. The maintainer line excludes the nuclear restorer gene but contains the fertile cytoplasmic gene (Chen and Liu, 2014). However, the restorer line preserves a functionally nuclear gene and with or without a fertile cytoplasmic gene (Chen and Liu, 2014). The pollens of both the maintainer and the restorer lines are fertile, so they can propagate by self-pollination. When the sterile line is used as the female parent, it can receive pollen from either the maintainer or restorer line and produce hybrid progeny. The maintainer line is used to cross with the male sterile line to reproduce the male sterile line, while the restorer line is used to cross with the male sterile line to produce hybrid progenies with heterosis to realize yield increase.

The *CMS/Rf* system has been exploited for hybrid seed production in plenty of crops such as maize, rice, wheat, rape, soybean, sorghum, carrot, sugar beet, sunflower, cotton, pepper, and petunia (Garcia, et al. 2019). Although this system has been successfully applied to soybean, the yield increase is still far away from that of rice and maize. One of the main reasons is the limited number of identified CMS lines, which heavily restricts the utilization of the three-line system in soybean hybrid seed production. In order to address this issue, the various cytoplasmic genes that produce MS phenotypes, along with their corresponding nuclear-encoded *restorer-of-fertility* genes, need to be identified urgently.

## Genic male sterility (GMS) system

GMS is controlled by nuclear genes without the influence of the cytoplasmic genome that are either insensitive or sensitive to environmental conditions, called genetically stable GMS and environment-sensitive genic male sterility (EGMS), respectively. In the case of EGMS, male fertility is often impressionable to different environmental conditions, including photoperiod (PGMS), temperature (TGMS), photoperiod and temperature (PTGMS), and humidity (HGMS) (Chen and Liu, 2014; Xue, et al. 2018; Abbas, et al. 2021). EGMS is regarded as an efficient genetic tool to develop two-line hybrids, since the need of a maintainer line can be eliminated, and the male sterile line can be propagated by self-pollination under specific conditions (Garcia, et al. 2019). In this system, almost every conventional inbred line is able to restore the fertility of the male-sterile line, and no negative effects related to sterility-inducing cytoplasm have been observed. Furthermore, genes of this system can be easily transferred to other genetic backgrounds (Yu, et al. 2015). EGMS has long been exploited to efficiently produce hybrid rice seeds within a two-line system consisting of a male-sterile line and a maintainer line. For instance, the first PGMS line Nongken 58S, discovered in japonica rice in 1973, is completely male sterile when grown under long-day conditions but male fertile when grown under short-day conditions (Shi 1985). While TGMS line Annong S-1, discovered in indica rice in 1988, is completely male sterile when grown at high temperatures but male fertile at low temperatures (Deng, et al. 1999).

Similar to the CMS system, the use of genetically stable GMS in plant breeding and hybrid seed production also involves three different lines, including a male sterile line, a maintainer line, and a restorer line. However, the reproduction of male sterile line in the GMS system presents a difficulty in that the segregation obtained in the cross with the maintainer line requires an additional step of identification and removal of 50% heterozygotes for hybrid seed production. Fortunately, the discovery of BMS systems, omitting the further cross and identification, has overcome this drawback, which including but not limited to seed production technology (SPT) (Wu, et al. 2016), and genome-editing technology (Song, et al. 2021).

The SPT platform consists of a transgenic maintainer line capable of maintaining and propagating non-transgenic nuclear male-sterile female lines for hybrid seed production (Qi, et al. 2020). The transgenic maintainer line includes three important elements: (i) the fertility restorer element, which can be used to complement the sterility phenotype of homozygous mutant. The most important component of this element is the functional GMS gene corresponding to the relevant mutant, which is often driven by a CaMV 35S or native promoter. (ii) The gametophyte devitalize element, which was used to destroy the pollen and cause the gametophyte sterility. The α-amylase is usually chosen and driven by an anther-specific promoter. (iii) The seed screening element, which can be used for seed separation by eyes or specific apparatus. The seed specific expressed promoter was used to activate the expression of fluorescence protein gene, such as the *mCherry* gene (Chang, et al. 2016; Wu, et al. 2016). The recessive genetic male-sterile mutant *ms45*, defective in the formation of outer pollen wall, was the first one used to establish the SPT system in maize (Wu, et al. 2016). Up to now, four fertility related genes, including rice *Oryza sativa No Pollen 1* (*OsNP1*) (Chang, et al. 2016), maize *MS44* (Fox, et al. 2017), *MS7* (Zhang, et al. 2018b), and *MS30* (An, et al. 2019) were successfully used in developing SPT systems, individually.

With the development of genome-editing technologies and the knowledge of the molecular genetics of male fertility genes, male sterile line can be created using CRISPR/Cas9 genome-editing technology, and is no longer limited by the naturally stable GMS mutants. Zhou et al. (2016) developed new “transgene clean” commercial TGMS lines in rice by knocking out *TMS5* via CRISPR/Cas9. Whereafter, Li et al. (2017) produced TGMS maize by targeted mutation of the maize homolog of rice *TMS5* (called *ZmTMS5*) using the CRISPR/Cas9 editing system. In addition, two rice reverse PGMS lines in *japonica* cultivars 9522 and JY5B were also generated by editing *Carbon Starved Anther* (*CSA*) gene using CRISPR/Cas9 (Li, et al. 2016). Furthermore, Qi et al. (2020) integrated CRISPR/Cas9 and SPT systems to generate *Zmms26* male sterility line in maize by co-transforming two independent vectors. In soybean, Chen et al. (2021) used targeted editing of *ABORTED MICROSPORES* (AMS) homologs by CRISPR/Cas9 technology for the first time to generate stable male-sterile lines in soybean. Since *GmAMS1* functions not only in the formation of the pollen walls, but also in the controlling the degradation of the soybean tapetum, targeted editing of *GmAMS1* resulted in a male-sterile phenotype. Recent advances in genomics and the emergence of multiple biotechnological approaches have revolutionized the field of crop breeding. However, identifying more male-sterility genes and elucidating the molecular mechanisms of male sterility in crops are prerequisites for hybrid crop breeding and heterosis exploitation.

## CMS system and hybrid breeding in soybean

Studies on soybean CMS started relatively late. The phenomenon of CMS was firstly observed by Davis (1985), while no further information about this mutant is available. The similar work of identification CMS line with wide cross between RNTED (Ru Nan Tian E Dan, *Glycine max*) and 5090035 (*Glycine soja*) was performed in China since 1983 by Sun et al., (1994a). It was not until 1985 that they found the high pollen abortion rate in (RNTED X 5090035) F_1_ at different environmental locations. In addition, they conducted reciprocal crossed in 1987 and uncovered that the pollen abortion rate of (RNTED X 5090035) was much higher than that of (5090035 X RNTED). All these results confirmed they found a real CMS line and named it with CMS-RN (Sun et al., 1994a).

Hereafter, another five CMS types were developed and reported in soybean (Table 1). Peng et al. (1994) found that ZD8319, also known as Zhongdou 19 (Sun, et al. 2003), processes CMS features, hereafter the derivative CMS lines from ZD8319 were attributed as CMS-ZD type, including of Zhongyou89B, M, ZA, W931A, FuCMS5A, SXCMS1A, SXCMS5A, and JLCMSZ9A (Zhao, et al. 1998; Zhang, et al. 1999a; Sun, et al. 2003; Dong, et al. 2012; Wang, et al. 2016; Dai, et al. 2017; Bai, et al. 2022). In addition, Zhao et al. (1998) confirmed that a *Glycine max* named XXT contained cytoplasmic male sterile gene, so this type of CMS was allocated as CMS-XX (Sun, et al. 2003). However, no further studies, such as cytological and functional analyses, have been performed for the CMS-XX line. Gai et al. (1995) discovered the cytoplasmic-nuclear male sterility phenomenon in the F_1_ of (N8855 X N2899, two *Glycine max* cultivars). Ding et al. (2002) further developed a CMS line NJCMS1A (maintainer line NJCMS1B) by back-crossing with the recurrent parent N2899 for another four successive generations. Since N8855 contributed the cytoplasmic gene, this new variety is called CMS-N8855 type. Subsequently, NJCMS3A (maintainer line NJCMS3B) derived from the cross between N21566 and N21249, exhibited different cytological characteristics when compared with NJCMS1A and NJCMS2A (N8855 X N1628, CMS-N8855), which was considered as a new CMS type, and was named CMS-N21566 type here after (Zhao and Gai, 2006). Furthermore, Nie et al. (2017) developed a CMS line NJCMS4A from the cross of N23661 X N23658. The mitochondrial markers and genome sequences analyses suggested that N23661 male sterile cytoplasm is distinguished from that of CMS-RN, CMS-ZD, CMS-N8855, and CMS-N21566 types, so CMS-N23661 was named as a new CMS type (Nie, et al. 2017).

**Table 1:** Characterized cytoplasmic male sterility type in soybean

Type of CMS	Soybean germplasms contributing male sterile cytoplasm	Representative CMS line	Fertility restorer locus/gene	References
CMS-RN	RNTED (Ru Nan Tian E Dan/167)	OA, YA, JLCMS5A, JLCMS8A, JLCMS9A, JLCMS14A, JLCMS29A, JLCMS31A, JLCMS47A, JLCMS82A, JLCMS84A, JLCMS89A, JLCMS101A JLCMS139A, JLCMS171A	*Rf1*, Chr. 16, *Glyma.16G161900*; *Rf3*, Chr. 9, *Glyma.09G171200*	(Sun et al., 1994a), (Sun et al., 1994b), (Sun, et al. 2001), (Zhang, et al. 2018a), (Zhang, et al. 2021), (Zhang, et al. 2018c), (Wang, et al. 2010), (Yan, et al. 2019), (Lin, et al. 2014), (Guo, et al. 2022), (Sun, et al. 2022), (Yang, et al. 2023)
CMS-ZD	ZD8319 (Zhongdou 19), Zhongyou 89B	M, ZA, W931A, FuCMS5A, SXCMS1A, SXCMS5A, JLCMSZ9A	*Rf*, Chr. 16, between BARCSOYSSR_16_1064 and BARCSOYSSR_16_1082; *Rf-m*, Chr. 16, between GmSSR1602 and GmSSR1610	(Zhao, et al. 1998), (Wang, et al. 2016), (Wang, et al. 2010), (Dong, et al. 2012), (Dai, et al. 2017), (Bai, et al. 2022), (Lin, et al. 2020), (Wang, et al. 2016), (Zhang, et al. 1999a), (Sun, et al. 2003)
CMS-XX	XXT	JLCMSPI9A	-	(Zhao, et al. 1998), (Sun, et al. 2003), (Lin, et al. 2020)
CMS-N8855	N8855	NJCMS1A, NJCMS2A	*Rf*, Chr. 16, *GmPPR576* (*Glyma.16G161900*)	(Ding, et al. 2002), (Wang, et al. 2021)
CMS-N21566	N21566	NJCMS3A	-	(Zhao and Gai, 2006)
CMS-N23661	N23661	NJCMS4A	-	(Nie, et al. 2017)

“_” represents not reported

To date, only the CMS system has been applied for the development of hybrid soybean. The CMS-RN is the earliest CMS type discovered and was first used in the three-line system used for hybrid breeding in soybean. Since the nuclear gene was replaced by the wild soybean 5090035, OA CMS line displayed the sprawling and shattering characteristics, which restricts its utilization for breeding (Zhang, et al. 1999a). Then Sun et al. (2001) used OA BC_3_ as the female parent to cross with the *Glycine max* and to create a new CMS line YA (maintainer line YB) in 1995, which brings the possibility to establish the three-line hybrid breeding system in soybean. The world's first commercially approved soybean hybrid HybSoy 1 was bred in China in 2002 through successive backcrossing (JLCMS9A X Jihui 1) (Zhao, et al. 2004). Taking advantage of this type of cytoplasm, more than five hundred pairs of CMS-RN lines and their corresponding maintainers have been bred, among which more than forty CMS lines with high sterility rate, high combining ability, and high outcrossing rate (called three-high CMS line) have been bred (Sun, et al. 2021). Totally, 42 commercialized soybean hybrid varieties have been developed in China (Table 2). Among the six different CMS types, CMS-RN was the most broadly used and totally 34 varieties were generated for spring soybean region in northern China (Table 2). In addition, the CMS-ZD type is often used in Huanghuai summer soybean region of China, and more than 50 pairs of CMS lines and the corresponding maintainer lines have been cultivated, but no three-high CMS line has been cultivated yet (Sun, et al. 2021). The hybrid soybean variety, named HybSoy 1 (Zhao, et al. 2004) and Zayoudou No.1 (Zayoudou 1) (Zhang, et al. 2007), increased the grain yield of soybean up to nearly 20% (Table 2).

**Table 2:** Hybrid soybean production with CMS system in China

No.	Name	Year of variety approval	Female parent	Male parent	Type of CMS	Yield increase compared with CK in production test (%)	CK variety	Oil (%)	Protein (%)	Oil and protein (%)
1	HybSoy 1	2002	JLCMS9A	JLR1	CMS-RN	20.8	Jilin 30	21.09	39.19	60.28
2	Zayoudou 1	2004	W931A	WR016	CMS-ZD	19.1	Zhongdou 20	18.96	43.56	62.52
3	HybSoy 2	2006	JLCMS47A	JLR2	CMS-RN	14.3	Jilin 30	20.54	40.75	61.29
4	HybSoy 3	2009	JLCMS8A	JLR9	CMS-RN	2.8	Heinong 38	20.84	40.54	61.38
5	Fuzajiaodou 1	2010	FuCMS5A	Fuhui 6	CMS-ZD	13.3	Zhongdou 20	19.43	44.65	64.08
6	Zayoudou 2	2010	W931A	WR99071	CMS-ZD	0.7	Zhongdou 20	18.80	44.12	62.92
7	HybSoy 4	2010	JLCMS47A	JLR83	CMS-RN	6.1	Heinong 38	19.57	40.48	60.05
8	HybSoy 5	2011	JLCMS84A	JLR1	CMS-RN	19.7	Jiunong 21	22.25	38.79	61.04
9	Jiyu 606	2013	JLCMS47A	JLR100	CMS-RN	13.5	Jiyu 47	21.51	40.11	61.62
10	Jiyu 607	2013	JLCMS14A	JLR83	CMS-RN	5.1	Jiyu 47	22.22	39.30	61.52
11	Jindou 48	2014	PZMS-1-1	ZH-21-B-5	CMS-RN	15.2	Jindou 19	19.89	36.99	56.88
12	Jiyu 608	2014	JLCMS84A	JLR113	CMS-RN	3.8	Jiyu 47	23.04	37.22	60.26
13	Jiyu 609	2015	JLCMS103A	JLR102	CMS-RN	2.2	Suinong 28	22.54	37.52	60.06
14	Fuzajiaodou 2	2016	FuCMS5A	Fuhui 9	CMS-ZD	7.3	Zhonghuang 13	18.99	46.86	65.85
15	Zayoudou 3	2016	W018A	M0901	CMS-ZD	6.4	Zhonghuang 13	18.81	46.06	64.87
16	Jiyu 610	2016	JLCMS128A	JLR98	CMS-RN	9.4	Suinong 28	21.15	37.32	58.47
17	Jiyu 611	2016	JLCMS147A	JLR113	CMS-RN	15.0	Jiyu 47	21.47	38.67	60.14
18	Jiyu 612	2017	JLCMS57A	JLR9	CMS-RN	4.4	Jiyu 72	20.92	42.07	62.99
19	Youshidou-A-5	2019	JLSXCMS1	Zhong 119-99	CMS-RN	12.1	Jindou 19	20.96	42.10	63.06
20	Jiyu 626	2019	JLCMS230A	JLR9	CMS-RN	11.4	Jiyu 72	20.19	40.68	60.87
21	Jiyu 627	2019	JLCMS179A	JLR9	CMS-RN	7.8	Jiyu 86	20.74	40.97	61.71
22	Jiyu 635	2019	JLCMS34A	JLR300	CMS-RN	14.8	Hejiao 02–69	22.71	36.27	58.98
23	Jiyu 639	2019	JLCMS191A	JLR403	CMS-RN	13.1	Hefen 55	23.78	37.04	60.82
24	Jiaji 1	2019	JLCMS178A	JLR124	CMS-RN	16.0	Hefen 55	22.15	40.46	62.61
25	Fudou 123	2020	FuCMS5A	Fuhui 5	CMS-ZD	4.9	Zhonghuang 13	18.15	44.96	63.11
26	Wanzadou 5	2020	W1101A	R1312	CMS-ZD	6.1	Zhonghuang 13	19.34	42.53	61.87
27	Jiyu 637	2020	JLCMS210A	JLR209	CMS-RN	8.3	Jiyu 86	20.19	41.70	61.89
28	Jiyu 641	2020	JLCMS191A	JLR158	CMS-RN	3.3	Hejiao 02–69	22.43	38.58	61.01
29	Jiyu 643	2020	JLCMS212A	JLR346	CMS-RN	11.2	Hejiao 02–69	21.69	38.14	59.83
30	Jiyu 647	2020	JLCMS5A	JLR2	CMS-RN	18.3	Hejiao 02–69	19.12	42.52	61.64
31	Jinong H1	2020	JLCMS254A	JLR192	CMS-RN	19.8	Hejiao 02–69	21.93	38.17	60.10
32	Jiyu 633	2020	JLCMS204A	JLR230	CMS-RN	14.0	Hefeng 50	20.44	42.78	63.22
33	Jindou 51	2021	NJCMS3A	C-19	CMS-N21566	12.3	Jindou 19	20.83	40.81	61.64
34	Jindou 52	2021	H3A	Laopinzhong 4	CMS-RN	11.9	Fendou 78	19.92	43.64	63.56
35	Jiyu 653	2021	JLCMS242A	JLR300	CMS-RN	9.6	Hejiao 02–69	22.89	37.68	60.57
36	Jiyu 654	2021	JLCMS234A	JLR13	CMS-RN	9.2	Hejiao 02–69	22.75	36.64	59.39
37	Jiyu 660	2021	JLCMS204A	JLR419	CMS-RN	8.0	Jiyu 86	21.42	41.39	62.81
38	Jiyu 645	2021	JLCMS234A	JLR9	CMS-RN	11.6	Suinong 26	20.12	44.77	64.89
39	Jinong H2	2021	JLCMS212A	JLR414	CMS-RN	9.6	Hejiao 02–69	22.91	38.86	61.77
40	Jiyu 649	2022	JLCMS209A	JLR158	CMS-RN	12.5	Hejiao 02–69	22.04	37.53	59.57
41	Jiyu 667	2022	JLCMS164A	JLR227	CMS-RN	6.4	Jiyu 86	21.08	39.76	60.84
42	Jiyu 668	2022	JLCMS247A	JLR227	CMS-RN	12.7	Hejiao 02–69	22.94	35.39	58.33

CK represents control. Data source: relevant variety approval reports

However, the cytological observations and functional analysis have lagged behind the applied research. Pollen abortion in male sterile lines may occur throughout the reproductive process, and the patterns of abortion varies greatly. A few cytological studies of the soybean CMS lines have been performed, and the studies mainly focused on the CMS-N8855, CMS-N21566, and CMS-ZD types. Ding et al. (2001) reported that the pollen abortion of NJCMS1A (CMS-N8855) occurred at the stage of binucleate pollen. In addition, Fan (2003) observed that the pollen abortion of NJCMS1A occurred at the stages of the microspore mother cells (MMCs), tetrads, uninucleate microspores and binucleate pollens, but most occurred at the early binucleate pollen stage. However, the microspore abortion of another CMS-N8855 type line NJCMS2A was mainly happened at the late uninucleate stage (Fan 2003). In addition, Zhao and Gai (2006) found that the microspore abortion of NJCMS3A (CMS-N21566) was mainly at the middle uninucleate stage, which was also confirmed by Fan (2003). Furthermore, Ren (2005) found that the pollen morphology of CMS line W931A (Zhongyou 89B X W206, CMS-ZD) was different from that of maintainer line W931B. The normal pollen grains from W931B were full, the surface of which was clearly visible, and the pollen apertures were easy to identify. However, the surface of the pollen grains from the CMS line W931A was blurred and shriveled, the pollen apertures could not be distinguished, and the size of the pollen grains was smaller than W931B.

The three-line system depends on the ability of the fertile restorer gene (*Rf*) in the restorer line to restore the CMS line’s fertility. With the development of the next-generation sequencing and the CRISPR/Cas9 technologies, multiple *Rf* genes will be identified in soybean, which will speed up the development of the three-line system in soybean. A set of works have been conducted for identifying *Rf* gene of CMS line, especially for CMS-RN type (Guo, et al. 2022). To narrow down the candidate region for *Rf1* gene for CMS-RN, Guo et al. (2022) constructed an F_2_ population by crossing JLCMS204A with JLR230 (restorer line), and the gene was located between the marker dCAPS-1 and BARCSOYSSR_16_1076., A recent study identified *Glyma.16G161900* as the candidate gene of *Rf1* (Yang, et al. 2023). In addition, *Glyma.09G171200*, encoding a pentatricopeptide repeat (PRR) protein, was confirmed as the candidate gene of another *Rf3* gene for CMS-RN (Sun, et al. 2022). In addition, the *Rf* gene of CMS-ZD type was located to the marker BARCSOYSSR_16_1064 and BARCSOYSSR_16_1082 on Chr. 16 (Dong, et al. 2012). Another *Rf-m* gene of CMS-ZD allocating on Chr. 16 was identified between GmSSR1602 and GmSSR1610 (Wang, et al. 2016). Furthermore, another *PPR* gene (*GmPPR576*, *Glyma.16G161900*) was identified as the candidate *Rf* gene of CMS-N8855 type (Wang, et al. 2021), which was consistent with that of *Rf1* gene (Yang, et al. 2023). Four *Rf* genes for CMS-RN, CMS-ZD and CMS-N8855 were closely distributed on Chr. 16 with a close region (Dong, et al. 2012; Wang, et al. 2016; Wang, et al. 2021; Guo, et al. 2022), whether they were controlled by the same gene needs to be further verified.

In addition, due to the lack of systematically cytological observation and the inconsistent cytological phenotype even for the same CMS type, for example, the CMS-N8855 line, whether the different CMS types are really distinguished from each other should be confirmed (Ding, et al. 2001; Fan 2003). Furthermore, an unusual phenomenon also happened that the same maintainer and restorer line can maintain and restore different CMS type, viz. YA (CMS-RN) and ZA (CMS-ZD) (Zhao, et al. 1998). Considering the contradictions, we cannot rule out the possibility that the six classified CMS types may not be completely different from each other.

## GMS system in soybean

The first report of GMS line in soybean was published in 1928, the mutant *st1* was both male and female sterile caused by abnormal chromosome association, which was controlled by a single recessive gene (Owen 1928). To date, approximately 30 GMS lines have been identified in soybean (Table 3). According to the phenotypic characteristics, *fs1fs2* (Johns and Palmer, 1982) and *ft* (transformed flower) (Singh and Jha, 1978) belong to the structural MS, the others belong to sporogenous MS, and no functional MS has been reported in soybean.

**Table 3:** The main GMS locus in soybean

No.	GMS name	Gene	Chr.	Marker	MS type	Phenotypic characteristic	Genetic characteristic	Mutation type	References
1	*ms3*	*Glyma.02G107600*	2	-	Photesensitive male sterile, female fertile	sporogenous	Sing-recessive gene	Natural variation	(Chaudhari and Davis, 1977), (Palmer, et al. 1980), (Hou, et al. 2022)
2	*88-428-BY*	-	-	-	Photesensitive male sterile, female fertile	sporogenous	Sing-recessive gene	Natural variation	(Wei 1991), (Wang, et al. 2004)
3	*ms8*	-	7	Sat_389, telomere	Temperature-sensitive male sterile, female sterile	sporogenous	Sing-recessive gene	Natural variation	(Palmer 2000), (Frasch, et al. 2011)
4	*ms9*	-	3	Satt521, Satt237	Temperature-sensitive male sterile, female sterile	sporogenous	Sing-recessive gene	Natural variation	(Palmer 2000), (Cervantes-Martinez, et al. 2007)
5	*msp*	-	2	GMES4176, Sat_069	Temperature-sensitive male sterile, female sterile	sporogenous	Sing-recessive gene	Natural variation	(Frasch, et al. 2011)
6	*st1*	-	-	-	Male sterile, female sterile	sporogenous	Sing-recessive gene	Natural variation	(Owen 1928), (Yan, et al. 2019)
7	*st2*	-	11	BARCSOYSSR_11_122, BARCSOYSSR_11_137	Male sterile, female sterile	sporogenous	Sing-recessive gene	Natural variation	(Hadley and Starnes, 1964), (Speth, et al. 2015)
8	*st3*	-	-	-	Male sterile, female sterile	sporogenous	Sing-recessive gene	Natural variation	(Hadley and Starnes, 1964)
9	*st4*	-	1	Satt436, Satt468	Male sterile, female sterile	sporogenous	Sing-recessive gene	Natural variation	(Palmer 1974), (Speth, et al. 2015)
10	*st5*	-	13	Satt030, Satt146	Male sterile, female sterile	sporogenous	Sing-recessive gene	Natural variation	(Palmer and Kaul, 1983), (Speth, et al. 2015)
11	*st6*	-	14	BARCSOYSSR_14_84, BARCSOYSSR_14_109	Male sterile, female sterile	sporogenous	Sing-recessive gene	Natural variation	(Skorupska and Palmer, 1990), (Speth, et al. 2015)
12	*st7*	-	2	Satg001, telomere	Male sterile, female sterile	sporogenous	Sing-recessive gene	Natural variation	(Jin, et al. 1997), (Speth, et al. 2015)
13	*st8*	-	16	E107, Satt132	Male sterile, female sterile	sporogenous	Sing-recessive gene	Natural variation	(Palmer and Horner, 2000), (Kato and Palmer, 2003)
14	*NJS-1H*	-	-	-	Male sterile, partial female sterile	sporogenous	Sing-recessive gene	Chemical mutagenesis	(Li, et al. 2010)
15	*D8804-7*	-	-	-	Partial male sterile, partial female sterile	sporogenous	Sing-recessive gene	Introduction of exogenous DNA	(Zhao, et al. 1995)
16	*fs1fs2*	-	-	-	Partial male sterile, partial female sterile	structural	Duplicate-recessive gene	Natural variation	(Johns and Palmer, 1982)
17	*ms1*	*Glyma.13G114200*	13	-	Male sterile, female fertile	sporogenous	Sing-recessive gene	Natural variation	(Brim and Young, 1971), (Palmer, et al. 1978), (Albertsen and Palmer, 1979), (Fang, et al. 2021), (Jiang, et al. 2021), (Nadeem, et al. 2021)
18	*ms2*	-	10	Sat_190, Scaa001	Male sterile, female fertile	sporogenous	Sing-recessive gene	Natural variation	(Graybosch, et al. 1984), (Graybosch and Palmer, 1985), (Cervantes-Martinez, et al. 2007)
19	*ms4*	*Glyma.02G243200*	2	-	Male sterile, female fertile	sporogenous	Sing-recessive gene	Natural variation	(Palmer 1979), (Delannay and Palmer, 1982), (Thu, et al. 2019)
20	*ms5*	-	-	-	Male sterile, female fertile	sporogenous	Sing-recessive gene	Physical mutagenesis	(Buss 1983)
21	*ms6*	*Glyma.13G066600*	13	-	Male sterile, female fertile	sporogenous	Sing-recessive gene	Natural variation	(Skorupska and Palmer, 1989), (Ilarslan, et al. 1999), (Yu, et al. 2021)
22	*ms7*	-	-	-	Male sterile, female fertile	sporogenous	Sing-recessive gene	Natural variation	(Palmer 2000)
23	*ms12*	*Glyma.10G117000*	10	centromere	Male sterile, female fertile	sporogenous	Sing-recessive gene	Chemical mutagenesis	(Zhang 2019)
24	*NJ89-1*	-			Male sterile, female fertile	sporogenous	Sing-recessive gene	Natural variation	(Ma, et al. 1993), (Yang, et al. 2003)
25	*msMOS*	-	2	Satt157, Satt698	Male sterile, female fertile	sporogenous	Sing-recessive gene	Natural variation	(Jin, et al. 1997), (Cervantes-Martinez, et al. 2009)
26	*ms*_*NJ*_	-	10	BARCSOYSSR_10_794, BARCSOYSSR_10_819	Male sterile, female fertile	sporogenous	Sing-recessive gene	Natural variation	(Nie, et al. 2019)
27	*N7241S*	-	-	-	Male sterile, female fertile	sporogenous	Single-dominant gene	Natural variation	(Zhao, et al. 2005)
28	*Wh921*	-	-	-	Male sterile, female fertile	sporogenous	Sing-recessive gene	Natural variation	(Zhang, et al. 1999b)
29	*mst-M*	-	13	W1, Satt516	Male sterile, female fertile	sporogenous	Sing-recessive gene	Natural variation	(Zhao, et al. 2019)
30	*ft*	-	-	-	Male sterile, female fertile	structural	Sing-recessive gene	Physical mutagenesis	(Singh and Jha, 1978)

This table is modified and updated from the recent review (Li, et al. 2019), “_” represents not reported

Two PGMS including *ms3* (Chaudhari and Davis, 1977) and *88-428-BY* (Wei 1991) and three TGMS including *ms8* (Palmer 2000), *ms9* (Palmer 2000), and *msp* (Stelly and Palmer, 1980) have already been reported. In addition, *st1*-*st8*, *NJS-1H*, *D8804-7*, and *fs1fs2* mutants were both male and female sterile (Owen 1928; Hadley and Starnes, 1964; Palmer 1974; Johns and Palmer, 1982; Palmer and Kaul, 1983; Skorupska and Palmer, 1990; Zhao, et al. 1995; Ilarsian, et al. 1997; Palmer and Horner, 2000; Kato and Palmer, 2003; Li, et al. 2010; Speth, et al. 2015). The *ms1* was the first GMS line that showed male sterile and female fertile phenotype in soybean (Brim and Young, 1971). In addition, *ms2*, *ms4*-*ms7*, *ms12*, *MJ89-1*, *msMOS*, *ms*_*NJ*_, *N7241S*, *Wh921*, *mst-M*, and *ft* also belong to the male sterile and female fertile category (Singh and Jha, 1978; Palmer 1979; Buss 1983; Graybosch, et al. 1984; Graybosch and Palmer, 1985; Skorupska and Palmer, 1989; Ma, et al. 1993; Jin, et al. 1997; Zhang, et al. 1999b; Palmer 2000; Zhao, et al. 2005; Zhang 2019; Zhao, et al. 2019).

Although five PGMS and TGMS lines have been identified, so far, only *MS3* (*Glyma.02G107600*), encoding a plant homeodomain (PHD) protein, has been identified (Hou, et al. 2022). The fertility of mutant *ms3* mutant can restore under long-day conditions, thus the mutant could be used to create a new, more stable photoperiod-sensitive genic male sterility line for two-line hybrid seed production in soybean. With the rapid development of BMS systems in rice and maize, more and more attempts have been made in soybean. The 13 GMS lines (*ms1*, *ms2*, *ms4-ms7*, *ms12*, *NJ89-1*, *msMOS*, *ms*_*NJ*_, *N7241S*, *Wh921*, and *mst-M*) displaying male sterile and female fertile phenotypes are suitable for exploiting this new technology in soybean. In order to make this design a reality, a large number of of works have been performed to explore the candidate sterile genes for these GMS mutants. *MS4* (*Glyma.02G243200*) is the first GMS gene that has been discovered in soybean by fine mapping, which encodes a MALE MEIOCYTE DEATH 1 (MMD1) protein (Thu, et al. 2019). The functional confirmation of *MS4* in regulating male fertility was conducted by heterologous expression in *Arabidopsis mmd1* mutant (Thu, et al. 2019). Subsequently, the function of *MS12* (*Glyma.10G117000*) was also confirmed by QTL mapping and functional complementation of soybean gene in *Arabidopsis cdc20.2* (cell division cycle 20.2) mutant (Zhang 2019). In 2021, both *MS1* (*Glyma.13G114200*) and *MS6* (*Glyma.13G066600*) have been identified by fine mapping (Fang, et al. 2021; Jiang, et al. 2021; Nadeem, et al. 2021; Yu, et al. 2021). *MS6* encodes a Tapetal Development and Functional 1 (TDF1) protein, a R2R3 MYB transcription factor, and predominantly expressed in anther, where it regulated the formation of pollen grain (Yu, et al. 2021). *MS1*, encoding a NPK1-ACTIVATING KINESIN 2 (NACK2) protein, is essential for cell plate formation after cytokinesis by directly control of the phragmoplast expansion (Fang, et al. 2021). Identification and characterization of GMS genes will provide more options for building the BMS systems for hybrid soybean production.

## Challenges and prospects in the commercialization of hybrid soybean

Although more than 40 hybrid soybean varieties have been generated from the three-line hybrid system (cytoplasmic male sterility), the unstable sterility of MS line and the high cost of hybrid seed production constrained the large-scale application of heterosis in soybean, which makes the cultivation of hybrid soybean still has a long way to go. We believe that the three components are the keys to make hybrid soybean a commercial success:

### Identify the male sterile lines with high out-crossing rate

The out-crossing rate is the key determinant of hybrid seed production. Seed production has not been efficient and cost-effective for hybrid soybean. The main reason is that the mutations in causing the male sterility also very often have pleiotropic effects and lead to the defect in female function, which make the male sterile lines with low seed set. The identification of *ms1* locus revealed that the gene was highly expressed in style and ovary and may also function in megagametogenesis or embryo development in soybean (Fang, et al. 2021). Studies had focused on the outcrossing rate on male sterile plants, the most promising record was the *ms2* mutant, the outcrossing rate on male sterile plants was 74% of the self-pollinated plants (Carter, et al. 1986; Perez, et al. 2009). The feasible solution is to speed up the cloning of the causal gene for male sterile mutants that have good recorded with seed-set, and simultaneously generate new male sterile lines by genome editing to make the mutation only affect the male fertility and without any effects on female productivity and other growth habits.

Besides the finding of ideal male sterility lines from the genetic perspective, the structure changes of flower and reproductive organs, for examples, the stigma protruding beyond the anthers, more pollen grains, and nectaries produce more fluids and/or volatiles, could increase the opportunity for cross-pollination (Palmer, et al. 2001). Pollen grain from soybean is heavy and sticky and the insect-mediated pollination is still indispensable even when the soybean flower is opened. The improvement of techniques for hybrid seed production is equally important for the commercialization of hybrid soybean, including the management of insect pollinators for cross-pollination and the suitable environment for both pollinators and soybeans, etc (Palmer, et al. 2001; Garibaldi, et al. 2021).

### Incorporate genomic selection to precise guidance on hybridization combination

Breeding 4.0 has been considered the next revolution of maize breeding (Wallace, et al. 2018). Even though the soybean breeding program is still at the Breeding 2.0 to 3.0 stages with molecular markers and genomic data to complement phenotypic data, the high-quality graph-based soybean pan-genome and the low cost of genome sequencing will turn promise into practice (Liu, et al. 2020b). The genotypes of soybean germplasm lines will be collected using high-throughput genotyping approaches such as next-generation sequencing (NGS) and SNP array platforms. Genetic variations among soybean germplasm of different origins/sources will make the selection of superior hybrid cost-effective.

### Good understanding of the molecular mechanisms of anther development in soybean

Little is known about the biological processes and genes that regulate anther and pollen development in soybean. Like most (70%) angiosperms, soybean produces bicellular pollen. By contrast, rice and *Arabidopsis* both produce tricellular pollen, the biological significance of the evolution of these two types of pollen grains is still unclear (Williams, et al. 2014). Bicellular pollen undergoes mitotic division to form two sperm cells after germination; prior to anthesis, tricellular pollen forms a male germ unit (MGU) that develops rapidly, which may make tricellular pollen favored in angiosperms that demand rapid reproduction (Hackenberg and Twell, 2019). So, the knowledge of extensive studies of anther and pollen formation in *Arabidopsis* and rice should not be simply transferred to soybean. Taking advantage of the comparative transcriptome analysis, the uncovering of anther-specific genes, genetic networks, and hub genes in soybean anther development will provide important insights into the molecular events underlying soybean reproductive developmental processes, as well as valuable resources for the plant reproductive biology community in the areas of pollen evolution, pollination/fertilization, and hybrid breeding.

In summary, on-going and future research should consider the enhancement of hybrid seed production efficiency, and the long-term investment and commitment will definitely make the commercialization of hybrid soybean a reality.

## Data Availability

The tables are included in this article albertsen.
